# How to Treat Algodystrophy and Rheumatic Comorbidity in Myelofibrosis: Three Case Reports

**DOI:** 10.7759/cureus.28058

**Published:** 2022-08-16

**Authors:** Olga Magazzino, Tiziana Urbano, Salvatore Magnasco

**Affiliations:** 1 Hematology, University of Bari, Bari, ITA; 2 Hematology, Ospedale San Giuseppe Moscati, Taranto, ITA; 3 Oncology, Ospedale Santissima Annunziata, Taranto, ITA

**Keywords:** algodystrophy, cytokine, inflammation, pain, neridronate, ruxolitinib, myelofibrosis

## Abstract

Algodystrophy or complex regional pain syndrome is a chronic pain condition characterized by hyperalgesia and allodynia. Patients with algodystrophy present an amplified and persistent activation of the innate immune system, with subsequent proliferation of keratinocytes and release of proinflammatory cytokines including interleukin (IL)-6, IL-1β, and tumor necrosis factor-α (TNF-α). Chronic inflammation and increased levels of cytokines are observed also in Ph-negative myeloproliferative neoplasms, including polycythemia vera, essential thrombocythemia, and primary myelofibrosis. Chronic myeloid neoplasms are characterized by overproduction of one or more mature non-lymphoid cell lineages, with erythrocytosis, thrombocytosis, and/or myeloproliferation.

Three case reports described our experience in the treatment of algodystrophy and rheumatic conditions in patients with myelofibrosis; a literature search was also performed.

The first patient was a 58-year-old woman who suffered from chronic myeloproliferative neoplasm in myelofibrotic evolution, under treatment with ruxolitinib and pre-treated with hydroxyurea; she reported inflammatory pain, and swelling of the tibiotarsal joints bilaterally. She was treated with neridronate 2 mg/kg for four days and methotrexate 15 mg per os per week, achieving a clinical benefit. The second patient was a 63-year-old woman diagnosed with polycythemia vera evolving to myelofibrosis. She experienced pain and swelling of the left tibiotarsal joint and difficulty walking. A therapy with low-dose steroid per os and intramuscular clodronate was administered for four months, followed by methotrexate at 15 mg per week. After two months, tenosynovitis significantly improved, as supported by the evidence of improved bone edema of the left tibiotarsal joint revealed in the magnetic resonance imaging, and pain symptoms were clinically ameliorated. The third patient was a 70-year-old male patient affected by essential thrombocythemia with myelofibrotic evolution and a paraneoplastic polymyalgia rheumatica treated with steroids and currently in remission. The patient received ruxolitinib for about two years; after the first year of treatment, he experienced pain and swelling of the right tibiotarsal joint with difficulty in walking, with a consequent diagnosis of edema and tenosynovitis, as per algodystrophy. After consulting a rheumatologist, the patient received therapy with neridronate intramuscularly with clinical benefit.

As overlapping interactions and clinical manifestations between hematologic neoplasms and rheumatologic diseases exist, new clinical manifestations, such as algodystrophy, may emerge during myelofibrosis and need to be monitored in the long term by a multidisciplinary team.

## Introduction

Algodystrophy is a chronic pain condition characterized by hyperalgesia and allodynia that can develop after extremity trauma, infection, or surgery [1]. The main features of algodystrophy are abnormal tissue response to injury, sensitization of the peripheral and central nervous systems, inflammatory changes, and autonomic dysregulation [2].

Focusing on the underlying inflammatory process, the clinical course of algodystrophy consists of an acute or warm phase, in which pro-inflammatory modulators are released, and a chronic or cold phase, where keratinocytes, fibroblasts, and osteocytes are activated [2].

During the acute phase, the release of pro-inflammatory cytokines, including interleukin-6 (IL-6), IL-1β, and tumor necrosis factor-α (TNF-α) [3], triggers an immune cascade that results in histamine-induced vasodilation, causing the redness, swelling, pain, and warmth. These cytokines also activate the connective tissue, causing contractures [4], and alter bone metabolism by acting on osteoblasts and osteoclasts [5]. Then, during the chronic phase, rapid bone turnover, bone loss, and osteoporotic changes occur [5].

Some evidence in animal models and preclinical studies indicate that even autoimmunity plays a role in algodystrophy [6]. In mice treated with anti-CD20 and in mu-MT mice (lacking mature B cells), after fracture/cast immobilization, algodystrophy-like symptoms were less severe compared to wild-type mice that had undergone the same procedure; IgM deposition and complement activation were also observed in the skin and sciatic nerves of wild-type fracture/cast mice [7]. Furthermore, experiments using immunohistochemical techniques and fluorescence-assisted cell sorting (FACS) analysis identified sympathetic nervous system neurons as targets for autoantibodies in some patients with algodystrophy, with little evidence of such autoimmunity from patients with other types of peripheral neuropathy [8].

Algodystrophy shows a variable progression over time and early initiation of the therapy is mainly aimed at restoring limb functionality, decreasing pain, and improving the quality of life. To reach these goals, a multidisciplinary approach involving patient education, physical and occupational therapy, along with pharmacological and surgical interventions, is helpful.

Non-steroidal anti-inflammatory drugs (NSAID) and corticosteroids have been traditionally used to manage pain and inflammation of algodystrophy; furthermore, based on the positive results that emerged from small randomized clinical trials, bisphosphonates are also introduced in the treatment of algodystrophy [2]. Bisphosphonates can modulate inflammatory mediators, proliferation, and migration of bone marrow cells but their mechanism of action has not been accurately detailed. Over the past three decades, several case reports described positive results in controlling pain, local inflammation, functional disability, and improving the quality of life of patients, especially in patients with early disease [9]. A randomized trial compared the efficacy of neridronate versus placebo in patients with algodystrophy and showed a significant improvement in the indices of pain and quality of life [10]. A meta-analysis of four randomized clinical trials including a total of 181 patients showed a significant reduction of pain in patients with algodystrophy with bisphosphonates compared to placebo, demonstrating the efficacy and safety of bisphosphonates in the treatment of the disease [11].

As in algodystrophy, inflammation is considered one of the factors that contribute to the development and progression of Ph-negative myeloproliferative neoplasms (MPNs), including polycythemia vera (PV), essential thrombocythemia (ET), and primary myelofibrosis (PMF). 

Indeed, current evidence suggests that MPNs are chronic inflammatory conditions in addition to neoplastic disorders and that both processes contribute to the clinical manifestations and pathogenesis of the disease [12]. The relationship between inflammation and myeloproliferation is supported by the evidence that increased levels of circulating cytokines and chemokines and the accumulation of reactive oxygen species in chronic inflammatory states can lead to genetic instability, which may promote the development and progression of neoplasms [12].

In MPNs, hyperactivation of the Janus kinase/signal transducer and activator of transcription (JAK-STAT) signaling due to the activating mutation V617F in the Janus kinase (JAK) 2 gene is frequently observed; in polycythemia vera and essential thrombocythemia, the JAK2 mutation can sustain a condition of chronic inflammation, explaining the associated constitutional symptoms, thrombosis, and premature atherosclerosis observed in patients with these disorders [12].

As the activating mutation V617F is a driver mutation in MPNs and is present in approximately 50% of patients with myelofibrosis, ruxolitinib, a potent oral inhibitor of JAK1/2, was tested in patients with myelofibrosis to examine the potential clinical benefit of JAK inhibition in this patients [13]. In a phase 1/2 trial, ruxolitinib showed clinical benefits associated with a marked diminution of levels of circulating inflammatory cytokines [13]. Therapeutic JAK2 inhibition with ruxolitinib reduced plasma levels of multiple cytokines in patients with myelofibrosis within the first month of treatment, without reverting them, however, to the low levels seen in healthy control plasmas [14]. Therefore, ruxolitinib can provide a partial, but incomplete, reduction of inflammatory pathophysiology in myelofibrosis. Other drugs currently used in patients with MPNs are hydroxyurea, anagrelide, and interferon [15].

Considering the high similarity in the inflammatory pathogenesis underlying both algodystrophy and MPNs, it is expected that clinical manifestations of rheumatological disorders are not uncommon during hematological malignancies. 

In these case reports, we described our experience in the treatment of algodystrophy and rheumatic conditions in patients with myelofibrosis.

## Case presentation

Per the World Medical Association Declaration of Helsinki, all the data referring to the patients are published anonymously, without any details allowing re-identification of the patient. Informed consents were signed by the patient, as required by the law of the country.

Case 1

A 58-year-old female patient reported inflammatory pain and swelling of the tibiotarsal joints bilaterally (Table 1).

**Table 1:** Summary of rheumatologic symptoms, autoimmunity testing, and treatment ANA, Antinuclear antibodies; ENA, extractable nuclear antigen; RF, rheumatoid factor; Anti-CCP, anti-cyclic citrullinated peptide

Case	Rheumatologic symptoms	Autoimmunity (FR, ANA, ANTI-CCP, ENA)	Pharmacological treatment
1. Woman, 58-year-old	Pain and swelling of tibiotarsal joints	Negative	Neridronate + steroids methotrexate
2. Woman, 63-year-old	Pain and swelling of tibiotarsal joints and metacarpophalangeal joints	Negative	Clodronate + steroids + methotrexate
3. Man, 70-year-old	Pain and swelling of tibiotarsal joints, polymyalgia rheumatica	Negative	Neridronate + steroids

She suffered from chronic myeloproliferative neoplasm in myelofibrotic evolution, for which she was treated with ruxolitinib, after previous therapy with hydroxyurea.

The patient was initially referred to a physiatrist who suggested a course of rehabilitating therapy, without achieving significant clinical benefit. Then, she underwent magnetic resonance imaging (MRI) that showed an inhomogeneous hyperintensity of signal in the gradient echo short-TI inversion recovery (GE-STIR) sequences of the talus, related to intraspongiosus edema for algodystrophy; intra-articular effusion and intraspongiosus edema of the cuboid, calcaneus, and peroneal malleolus were also observed. Hyperintense signal in peroneal-calcaneal and peroneal-astragalic ligament was due to synovitis and minimal enthesopathy of the Achilles tendon was revealed. Therefore, the patient was invited to consult a rheumatologist, although in her clinical history neither previous episodes of arthralgia due to inflammation nor familiarity with psoriasis and inflammatory bowel disease were accounted for. Rheumatological biomarkers, including antinuclear antibodies (ANA), anti-extractable nuclear antigen (ENA) profile, rheumatoid factor (RF), and anti-cyclic citrullinated peptide (CCP) antibodies were negative. After the diagnosis of algodystrophy, she started 5 mg of prednisone and two cycles of intravenous neridronate 2 mg/kg for four days.

**Figure 1:**
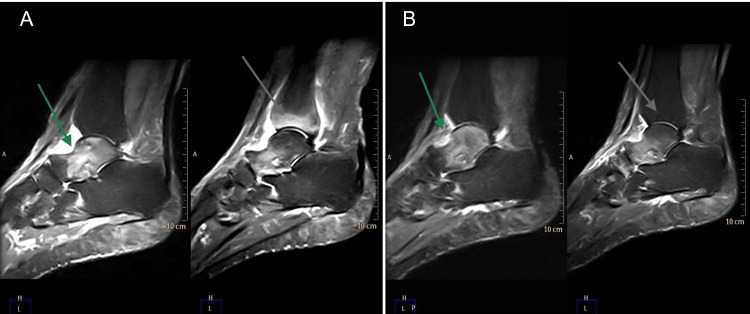
Magnetic resonance imaging of tibiotarsal joints before (A) and after (B) the treatment with neridronate, with improvement of bone edema (grey arrow) and synovitis (green arrow).

After the first cycle of infusions, the patient reported a clinical benefit, but tenosynovitis persisted. For this reason, methotrexate 15 mg per os per week was added to therapy, with monitoring of renal function and transaminases. Two months after methotrexate initiation, the patient maintained the clinical benefit, with a significant reduction of pain in the tibiotarsal joints. MRI consistently showed a reduction of intraspongiosus bone edema of the tibiotarsal joints bilaterally (Figure 1).

A bone marrow biopsy confirmed the hematological diagnosis of primary myelofibrosis (grade II-III fibrosis), with a cluster of differentiation (CD)3+/-; CD20-/+ phenotype.

Case 2

In 2009, a 63-year-old woman was diagnosed with polycythemia vera; the disease was previously treated with phlebotomy, hydroxyurea, and anagrelide, which was interrupted for tachycardia, and was evolving into myelofibrosis. The patient presented also osteoporosis with vertebral wedging of the second lumbar vertebra. In July 2020, pain and swelling of the left tibiotarsal joint appeared and the patient had difficulty in walking (Table 1). After performing an MRI of the left tibiotarsal joint that showed evidence of tenosynovitis and bone edema the patient was referred for rheumatology consultation. Rheumatological biomarkers ANA, anti-ENA, anti-CCP, and FR were negative and inflammatory indexes were normal; only alkaline phosphatase was slightly increased. Therapy with 5 mg of prednisone per os and intramuscular clodronate was started. After four months, the pain was improved, although synovitis of the tibiotarsal bilaterally persisted and tumefaction of II and III metacarpophalangeal joints bilaterally appeared. Methotrexate at 15 mg per week was administered, with monitoring of creatinine and liver cytolysis indexes. After two months, tenosynovitis and bone edema of the left tibiotarsal joint significantly improved, as revealed in the MRI control, along with clinical amelioration of the pain.

Case 3

A 70-year-old male patient was affected by essential thrombocythemia with myelofibrotic evolution. At the onset of hematological pathology, the patient presented also a paraneoplastic polymyalgia rheumatica in remission with steroids (Table 1). The patient was initially treated with hydroxyurea and, then with ruxolitinib for about two years. After the first year of treatment with ruxolitinib, he experienced pain and swelling of the right tibiotarsal joint with difficulty in walking. MRI detected bone edema and tenosynovitis, as per algodystrophy. After consulting a rheumatologist, the patient received therapy with intramuscular neridronate with clinical benefit.

## Discussion

Few data are currently available in the literature on the association between rheumatoid arthritis and MNP and are mainly case reports. A 57-year-old man with rheumatoid arthritis in remission was diagnosed with essential thrombocythemia for persistent thrombosis; the biopsy identified the presence of JAK2 V617F mutation [16]. A report described severe back pain in a patient with seronegative spondyloarthropathy who was successfully treated with methotrexate and NSAIDs. When the patient complained of recurrence of back pain despite the ongoing therapy, a computed tomography scan-driven vertebral biopsy was performed and showed a markedly hypercellular bone marrow with trilinear hyperplasia, along with a slightly delayed maturation of all lineages and some atypia of megakaryocytes. JAK2 V617F was also identified, thus supporting the presence of polycythemia vera [17]. These case reports described the potential presence of a hematological disease concomitant to rheumatoid arthritis, ankylosing spondylitis, or other seronegative spondyloarthropathies, that should be kept in mind to avoid delayed diagnosis and unnecessary or dangerous treatments.

A 32-year-old man with human leukocyte antigen B-27-positive ankylosing spondylitis and JAK2 mutated essential thrombocythemia was initially treated with methotrexate and anagrelide, achieving a gradual control of both diseases. Due to a hepatic lesion, methotrexate was switched to etanercept, maintaining the therapy with anagrelide. The concomitant treatment with etanercept and anagrelide resulted as feasible and led to significant clinical improvements, reductions in inflammation markers, and improvement of functional status, as measured by disease activity indices [18]. A 38-year-old woman with rheumatoid arthritis showed impeding digital gangrene that led to the diagnosis of essential thrombocytosis with JAK2 mutation. The patient was treated with hydroxyurea and aspirin and platelet count progressively ameliorated; however, this improvement was not persistent, since other gangrenes on the toe and nails developed [19]. A 62-year-old woman suffering from non-erosive peripheral arthritides with general health impairment and high acute-phase reactant levels; previously, she had experienced chronic polyarthralgia and thrombocytosis discovered nine years before. All immunological blood tests were negative. The patient was initially treated with corticosteroid and methotrexate which improved pain, swollen joint count, and systemic inflammation. However, her joints remained stiff and painful with two swollen wrists and persistent thrombocytosis. When an iliac bone marrow biopsy was performed and detected primary myelofibrosis, hydroxyurea 500 mg per day was started, with a rapid, complete and persistent clinical and biological remission. After six months, a new disease flare occurred, and the increase of hydroxyurea to 1500 mg per day allowed us to reach again the remission [20].

Our experience and all the cases reported in the literature highlighted the importance of a multidisciplinary approach to care for patients with myelofibrosis and algodystrophy or other rheumatoid conditions, involving both hematologists and rheumatologists. Further studies are warranted to better understand the potential association between hematological and rheumatoid diseases.

## Conclusions

Numerous pieces of evidence in the literature describe overlapping interactions and clinical manifestations between hematologic neoplasms and algodystrophy. The interaction between inflammatory cytokines, pro-oncogenic molecules expressed by immature neoplastic clones, and angiogenesis could contribute to the development of articular clinical manifestations during haematologic diseases. Hematological neoplasms require an aggressive pharmacological approach, which often involves multiple treatments with chemotherapeutic drugs, monoclonal antibodies, and JAK inhibitors such as ruxolitinib. New clinical manifestations, such as algodystrophy, may emerge and need to be monitored in the long term.
